# Prevalence of comorbidities in cases of Middle East respiratory syndrome coronavirus: a retrospective study

**DOI:** 10.1017/S0950268818002923

**Published:** 2018-11-05

**Authors:** F.Y. Alqahtani, F.S. Aleanizy, R. Ali El Hadi Mohamed, M. S. Alanazi, N. Mohamed, M. M. Alrasheed, N. Abanmy, T. Alhawassi

**Affiliations:** 1 Department of Pharmaceutics, College of Pharmacy, King Saud University, 22452 Riyadh 11495, Saudi Arabia; 2 College of Science, Princess Nourah Bint Abdulrahman University, Riyadh 12484, Saudi Arabia; 3 Federal Ministry of Health, Khartoum 303, Sudan; 4 Emergency medicine consultant, Emergency Department, Prince Mohamed Bin Abdulaziz Hospital, Ministry of Health, Riyadh 12455, Saudi Arabia; 5 College of Medicine, Princess Nourah bint Abdulrahman University, Riyadh 12484, Saudi Arabia; 6 Clinical Microbiology Department, Umeå University, Virology Unit, Sweden; 7 Department of Clinical Pharmacy, College of Pharmacy, King Saud University, 22452 Riyadh 11495, Saudi Arabia

**Keywords:** Comorbidities, middle east respiratory syndrome coronavirus, mortality

## Abstract

The Middle East respiratory syndrome coronavirus (MERS-CoV) is a life-threatening respiratory disease with a high case fatality rate; however, its risk factors remain unclear. We aimed to explore the influence of demographic factors, clinical manifestations and underlying comorbidities on mortality in MERS-CoV patients. Retrospective chart reviews were performed to identify all laboratory-confirmed cases of MERS-COV infection in Saudi Arabia that were reported to the Ministry of Health of Saudi Arabia between 23 April 2014 and 7 June 2016. Statistical analyses were conducted to assess the effect of sex, age, clinical presentation and comorbidities on mortality from MERS-CoV. A total of 281 confirmed MERS-CoV cases were identified: 167 (59.4%) patients were male and 55 (20%) died. Mortality predominantly occurred among Saudi nationals and older patients and was significantly associated with respiratory failure and shortness of breath. Of the 281 confirmed cases, 160 (56.9%) involved comorbidities, wherein diabetes mellitus, hypertension, ischemic heart disease, congestive heart failure, end-stage renal disease and chronic kidney disease were significantly associated with mortality from MERS-CoV and two or three comorbidities significantly affected the fatality rates from MERS-CoV. The findings of this study show that old age and the existence of underlying comorbidities significantly increase mortality from MERS-CoV.

## Introduction

The first case of Middle East respiratory syndrome coronavirus (MERS-CoV) was reported in Saudi Arabia in 2012 and the novel causative virus, MERS-CoV, was identified [1, 2]. To date, 2040 laboratory-confirmed cases of MERS-CoV infection have been reported to the World Health Organization [3] from 27 countries [3]. Most reported cases were from countries in the Middle East, among which 82% occurred in Saudi Arabia; other cases were reported from North Africa, Europe, the USA and East Asia, with the latter cases involving individuals who had travelled to the Middle East [3]. Owing to the high mortality rate and the lack of antiviral treatment or a preventative vaccine, MERS-CoV remains a major public health concern.

Coronaviruses are enveloped, positive-sense, single-stranded RNA viruses that belong to the subfamily Coronavirinae within the Nidovirales order, which are further subdivided into four genera: alpha, beta, gamma and delta. Human coronaviruses belong to the alpha and beta genera [4].

Phylogenetically, MERS-CoV belongs to the beta coronavirus lineage C, with a genotype that is very closely associated with bat coronaviruses from the same lineage, such as BtCoV-HKU4 and BtCoV-HKU5 [5]. MERS-CoV is considered as a zoonotic virus that can cause secondary infections in humans. While dromedary camels were recognized as the intermediate host of MERS-CoV, human-to-human transmission has been observed in community clusters [6–9], among hospital contacts [10, 11] and among healthcare workers [11], which raises concerns regarding its pandemic risk [11].

MERS-CoV is more prevalent in males [3, 10, 12, 13] and has a clinical spectrum ranging from asymptomatic to life-threatening, which includes acute respiratory distress syndrome, pneumonia, myocarditis and organ failure [10, 12, 13]. The case fatality rate of MERS-CoV ranges from 30% to 60% [13–17], in which severe disease presentation is correlated with pre-existing medical conditions such as diabetes, cardiovascular diseases, renal failure, obesity and immunodeficiency [13, 18]. Thus, the present paper aims to investigate the effect of comorbidities on the mortality rate of confirmed MERS-CoV cases in Saudi Arabia.

## Methods

### Data collection

All laboratory-confirmed MERS-CoV cases reported by the Saudi Ministry of Health to WHO between 23 April 2014 and 7 June 2016 were identified. Patient charts were reviewed for demographic information, mortality, comorbidities and clinical presentation.

### Case definition

A suspected case was defined as any instance of hospitalization for bilateral pneumonia and any one of the following clinical symptoms at admission: fever (>38 °C), cough, shortness of breath (SOB), sore throat, vomiting, diarrhoea, haemoptysis, chest pain and/or infection, respiratory failure, loss of consciousness, runny nose and any asymptomatic cases with a history of contact with confirmed symptomatic cases.

A confirmed case was a suspected case with a laboratory-confirmed MERS-CoV infection on the basis of positive real-time polymerase chain reaction (RT-PCR) results for MERS-CoV in swab samples collected by the Ministry of Health. Signed informed consent was obtained from patients or the legal guardians of minors for the use of their (coded) data for research purposes.

Asymptomatic cases were those with no reported symptoms at the time of a positive test recorded by a healthcare provider in the medical chart. However, these patients showed symptoms subsequently in the clinical course. In contrast, in symptomatic cases, patients reported their symptoms during hospital admission.

### Molecular testing

All RT-PCR tests were performed at the Saudi Ministry of Health MERS-CoV regional laboratory in Riyadh. Respiratory samples were obtained from all patients and were submitted to the regional laboratory for testing using primers that amplify both the upstream E protein (*upE*) and *ORF1a* genes. Samples that tested positive for both *upE* and *ORF1a* gene targets were considered as confirmed cases. Each patient was tested at least twice and each test was conducted on a different day.

### Statistical analyses

Statistical analyses were performed for determining significant differences using *t* tests, the *χ*^2^ test and Fisher's exact test where appropriate. The odds ratio (OR) and 95% confidence interval (CI) were obtained for each variable. Statistical analyses were performed using SPSS, version 21 software (IBM Corp., Armonk, NY, USA). A *P*-value < 0.05 was considered statistically significant.

## Results

A total of 281 confirmed MERS-CoV cases from Saudi Arabia were reported to the Saudi Ministry of Health from 23 April 2014 to 7 June 2016. Table 1 shows the distribution of the cases by sex, age, nationality, symptoms and fatality rates. The ratio of male to female cases was 1.45:1. Of the 281 confirmed cases, 55 (20%) died and the case fatality rate was higher in males than females, even though this difference was not statistically significant (67.3% *vs.* 32.7%; OR: 1.5, 95% CI 0.81–2.83; *P* = 0.221). Death predominantly occurred in Saudi citizens (*P* = 0.047) and in the group aged older than 60 years (*P* < 0.0001). The most prevalent clinical symptoms included fever (62%) and cough (54.1%), followed by SOB (41.3%; Table 2). Death among laboratory-confirmed cases of MERS-COV infection was significantly associated with a SOB and respiratory failure (*P* < 0.001 and *P* < 0.007, respectively; Table 2).

**Table 1.** Mortality in Middle East respiratory syndrome coronavirus confirmed cases in Saudi Arabia between 23 April 2014 and 7 June 2016

	Survival			OR (95% CI)	*P*-value
	Alive (*n* = 226) (%)	Died (*n* = 55) (%)	Total (*n* = 281) (%)		
Sex					
Male	130 (57.5)	37 (67.3)	167 (59.4)	1.517 (0.815–2.827)	0.187
Female	96 (42.5)	18 (32.7)	114 (40.6)		
Nationality					
Saudi	127 (56.2)	39 (70.9)	166 (59.1)*	1.900 (1.004–3.598)	0.047
Non-Saudi	99 (43.8)	16 (29.1)	115 (40.9)		
Symptoms					
Yes	187 (82.7)	51 (92.7)	238 (84.7)	2.6591 (0.907–7.788)	0.065
No	39 (17.3)	4 (7.3)	43 (15.3)		
Age group (year)					
<20	9 (4.0)	0 (0)	9 (3.2)	0.206 (0.011–3.598)	0.279
21–40	92 (40.7)	13 (23.6)	105 (37.4)	0.450 (0.229–0.886)	0.02
41–60	85 (37.6)	16 (29.1)	101 (35.9)	0.680 (0.358–1.292)	0.239
>61	40 (17.7)	26 (47.3)	66 (23.5)**	4.169 (2.220–7.827)	<0.0001

Data presented as *n* (%).

**Table 2.** Symptoms of Middle East respiratory syndrome coronavirus in confirmed cases at presentation

	Survival			OR (95% CI)	*P*-value
	Alive (*n* = 226) (%)	Died (*n* = 55) (%)	Total (*n* = 281) (%)		
Cough	122 (54.0)	30 (54.5)	152 (54.1)	1.023 (0.566 −1.848)	0.940
Fever	139 (61.5)	35 (63.6)	174 (62)	1.095 (0.594–2.018)	0.770
Diarrhoea	22 (9.7)	1 (1.8)	23 (8.2)	0.171 (0.022–1.302)	0.083
Shortness of breath	82 (36.3)	34 (61.8)	116 (41.3)	2.843 (1.548–5.221)	0.001*
Sore throat	22 (9.7)	0 (0)	22 (8)	0.081 (0.004–1.371)	0.08
Vomiting	10 (4.4)	4 (7.3)	14 (5)	1.6941 (0.510–5.619)	0.284
Chest pain	1 (0.4)	0 (0)	1 (0.4)	1.354 (0.054–33.698)	0.804
Runny nose	1 (0.4)	0 (0)	1 (0.4)	1.354 (0.054–33.698)	0.804
Respiratory failure	0 (0)	3 (5.5)	3 (1.1)	30.200 (1.536–593.628)	0.02*
Asymptomatic	39 (17.7)	4 (7.3)	43 (15.3)	0.376 (0.128–1.101)	0.074

*Significant *p* value.

Among the 281 cases, 160 (56.9%) patients had underlying comorbid conditions (Table 3).

**Table 3.** Number of comorbidities in relation to mortality from Middle East respiratory syndrome coronavirus infection

	Survival		OR (95% CI)	*P*-value
	Alive (*n* = 226)	Died (*n* = 55)		
Comorbidity				
Yes	114 (50.4%)	46 (83.6%)	5.021 (2.347–10.742)	<0.0001*
No	112 (49.6%)	9 (16.4%)		
Total number of comorbidities				
One	65 (28.8%)	17 (30.9%)	1.1081 (0.584–2.102)	0.7534
Two	32 (14.2%)	15 (27.3%)	5.833 (2.33–14.56)	0.0217*
Three	15 (6.6%)	13 (23.6%)	10.785 (1.930–9.818)	0.0004*
Four	2 (0.9%)	0 (0%)	0.809 (0.038–17.092)	0.8917
Five	0 (0%)	1 (1.8%)	12.4679 (0.501–310.273)	0.1239
Total number of comorbidities Mean ± s.d.	0.805 ± 0.974	1.655 ± 1.126		

*Significant *p* value.

Patients with comorbidities had a higher mortality risk compared with those without (83.6% *vs.* 16.4%, *P* < 0.0001). The number of comorbidities in relation to mortality is shown in Table 4. Diabetes mellitus (DM), hypertension (HTN), cardiac diseases, renal disease and bronchial asthma were the most frequent comorbid disorders (Table 4). Among the comorbidities, DM, HTN, ischemic heart disease (IHD), congestive heart failure (CHF), end-stage renal disease (ESRD) and chronic kidney disease (CKD) showed significant associations with fatality from MERS-CoV (OR 2.6 (1.4–4.9), OR 3.7 (2.02–6.9), OR 6.1 (2.1–17.2), OR 12.9 (1.3–127.3), OR 5.8 (1.2–26.8) and OR 5.2 (4.1–6.7), respectively; Table 4). In this regard, the prevalence of death in diabetic and hypertensive patients was similar (28 (50.9%) and 29 (52.7%)), respectively. Further analysis of those patients who had these comorbidities including DM, HTN, IHD, CHF, ESRD and CKD that contributed significantly to mortality from MERS-CoV showed no significant difference in mortality (*P* > 0.05) between symptomatic and asymptomatic groups (data not shown). Moreover, only four cases of asymptomatic patients died representing 1% of total 281 cases, three of these four were elderly aged more than 66-years-old with comorbidities (HTN and/or dementia) and one case aged 55-years-old and had additional multiple comorbidities (DM, HTN, COPD). Taking as presented in Table 4, the presence of two or three comorbidities significantly increased the risk of death from MERS-CoV infection (*P* < 0.0001).

**Table 4.** Comorbidities in confirmed cases of Middle East respiratory syndrome

	Survival		OR (95% CI)	*P*-value
	Alive (*n* = 226) (%)	Died (*n* = 55) (%)		
Diabetes mellitus	63 (27.9)	28 (50.9)	2.683 (1.468–4.905)	0.001*
Hypertension	52 (23.0)	29 (52.7)	3.732 (2.021–6.892)	<0.0001*
CVA	4 (1.8)	1 (1.8)	1.028 (0.113–9.382)	0.666
IHD	7 (3.1)	9 (16.4)	6.121 (2.169–17.277)	0.001*
HF	1 (0.4)	0 (0)	1.354 (0.0544–33.6980)	0.804
CHF	1 (0.4)	3 (5.5)	12.981 (1.324–127.313)	0.025*
Mitral + Aortic valve replacement	0 (0)	1 (1.8)	12.467 (0.5010–310.2738)	0.196
COPD	1 (0.4)	1 (1.8)	4.167 (0.257–67.680)	0.354
Lung cancer	1 (0.4)	0 (0)	1.354 (0.0544–33.6980)	0.804
Chronic Lung Disease	1 (0.4)	0 (0)	1.354 (0.0544–33.6980)	0.804
Pneumonia	4 (1.8)	1 (1.8)	1.028 (0.113–9.382)	0.666
B. Asthma	24 (10.6)	3 (5.5)	0.486 (0.141–1.675)	0.244
Myasthenia Gravis	0 (0)	1 (1.8)	12.467 (0.5010–310.2738)	0.196
Colon cancer	1 (0.4)	0 (0)	1.354 (0.0544–33.6980)	0.804
Renal failure	1 (0.4)	0 (0)	1.354 (0.0544–33.6980)	0.804
End-stage renal disease	3 (1.3)	4 (7.3)	5.830 (1.266–26.858)	0.029*
Chronic kidney disease	0 (0)	2 (3.6)	5.264 (4.131–6.707)	0.038*
Myeloma	1 (0.4)	0 (0)	1.354 (0.0544–33.6980)	0.804
Gout	1 (0.4)	0 (0)	1.354 (0.0544–33.6980)	0.804
Anaemia	0 (0)	1 (1.8)	12.467 (0.5010–310.2738)	0.196
Hodgkin's Lymphoma	1 (0.4)	0 (0)	1.354 (0.0544–33.6980)	0.804
Parkinsonism	1 (0.4)	0 (0)	1.354 (0.0544–33.6980)	0.804
Liver carcinoma	1 (0.4)	0 (0)	1.354 (0.0544–33.6980)	0.804
Liver Cirrhosis	1 (0.4)	2 (3.6)	8.491 (0.756–95.392)	0.099
Hypothyroidism	2 (0.9)	2 (3.6)	4.226 (0.5819–30.6946)	0.154
Sickle cell anaemia	1 (0.4)	0 (0)	1.354 (0.0544–33.6980)	0.804
Tuberculosis	2 (0.9)	0 (0)	1.246 (1.175–1.320)	0.646

*Significant *p* value.

## Discussion

In the present study, the mortality rate was 20% among the 281 confirmed cases of MERS-CoV infection in Saudi Arabia between 23 April 2014 and 7 June 2016. Mortality was higher in male patients, with a male-to-female ratio of 1.45:1. The predominance of male cases is consistent with the findings reported in previous studies in Saudi Arabia and South Korea [19, 20], which may be associated with cultural or occupational behaviours in males that increase the risk of infection. Saudi citizens showed significantly higher mortality than non-Saudi citizens. The demographic features of the laboratory-confirmed cases of MERS-COV infection in the current study showed that only nine patients were aged <20 years; 73.2% were aged 21–60 years and 23.5% were aged >61 years. These findings are in agreement with those of previous studies reported in Saudi Arabia [14, 21–23]. In addition, the higher mortality rate among elderly patients (>61 years) is consistent with the rate reported in a previous study [24]. A possible explanation for this may be age-associated immunosenescence, which results in a suboptimal immune response in these individuals following exposure to MERS-CoV [25] and the possible interference of underlying comorbidities in elderly patients cannot be ruled out. The clinical presentations of MERS-CoV cases observed in this study have similar symptomatology and frequency to those previously reported [10, 13, 18]. Remarkably, among the noted symptoms, SOB and respiratory failure were significantly associated with higher mortality.

In this study, chronic conditions such as DM, HTN, IHD, CHF and renal disease significantly influenced the severity of MERS-CoV. The observed increase in the mortality rate in MERS-CoV patients with underlying chronic conditions corroborates the findings of earlier studies in Saudi Arabia [13, 18]. This is also in line with the findings for other respiratory diseases such as influenza [26], influenza A H1N1 [27, 28] and SARS [29]. A recent study found that obesity, cardiovascular, hypertension and neuromuscular disease were strongly associated with severe pandemic influenza [26]. In SARS, comorbidities such as immunological, neurological, metabolic and dermatologic diseases were strongly associated with the disease [29]. These conditions are known to weaken the host's innate and humoral immune systems, thereby limiting their ability to counteract any new infection [30].

The present study has some limitations. First, although all confirmed MERS-CoV cases were notified to the Saudi Ministry of Health from different regions and hospitals in Saudi Arabia. However, we cannot rule out the possibility that some cases have gone undetected. Second, comorbidity data for admissions without MERS-CoV infection were unavailable.

In conclusion, the present study has shown that old age and the presence of comorbidities are associated with adverse outcomes in MERS-CoV patients, including increased mortality rates.
